# Syncope and In-Hospital Mortality in Pulmonary Embolism: Insights from 300 Patients

**DOI:** 10.3390/life15091437

**Published:** 2025-09-13

**Authors:** Corina Cinezan, Camelia Bianca Rus

**Affiliations:** 1 Department of Medical Disciplines, Faculty of Medicine and Pharmacy, University of Oradea, 410073 Oradea, Romania; 2 Clinical County Emergency Hospital Bihor, 410169 Oradea, Romania; 3 Doctoral School of Biological and Biomedical Sciences, University of Oradea, 410087 Oradea, Romania

**Keywords:** pulmonary embolism, syncope, in-hospital mortality, right ventricular dysfunction, prognostic marker, risk stratification, reperfusion therapy

## Abstract

**Background**: Pulmonary embolism (PE) is a major cause of cardiovascular mortality, with heterogeneous presentation. Syncope, reported in 10–20% of cases, may indicate severe hemodynamic compromise, but its independent prognostic role remains uncertain. **Methods**: We retrospectively analyzed 300 patients admitted with acute PE confirmed by computed tomography pulmonary angiography between January 2022 and December 2024. Patients were stratified according to the presence of syncope at admission. Clinical, laboratory, and echocardiographic findings and outcomes were compared. Logistic regression was used to identify independent predictors of in-hospital mortality. The present study is a single-center, retrospective analysis. **Results**: Syncope occurred in 48 patients (16%). Compared with those without syncope, these patients had higher rates of right ventricular dysfunction (68.7% vs. 32.5%, *p* = 0.002), elevated troponin (75% vs. 44%, *p* = 0.01), hypotension (29% vs. 8%, *p* < 0.001), Intensive Care Unit (ICU) admission (56% vs. 22%, *p* < 0.001), and thrombolysis (19% vs. 8%, *p* = 0.03). In-hospital mortality was markedly higher in the syncope group (37.5% vs. 7.1%, *p* < 0.001). Multivariate analysis confirmed syncope as an independent predictor of mortality (OR 4.2, 95% CI 2.1–8.4, *p* < 0.001), alongside right ventricular dysfunction and elevated troponin. **Conclusions**: Syncope at presentation is a simple but powerful clinical marker of high-risk PE and should prompt intensive monitoring and consideration of early reperfusion therapy.

## 1. Introduction

Pulmonary embolism (PE) is a common and potentially fatal cardiovascular emergency caused by thromboembolic obstruction of the pulmonary arterial circulation. It represents the third most frequent acute cardiovascular syndrome after myocardial infarction and stroke, with an estimated annual incidence of 60–70 cases per 100,000 population in Europe and North America. Despite advances in diagnostic imaging and therapeutic strategies, PE continues to carry a considerable burden of morbidity and mortality, with reported in-hospital mortality rates ranging from 8% to 15% depending on clinical severity and comorbidities [1,2,3].

The clinical presentation of PE is notoriously heterogeneous, ranging from asymptomatic or mild dyspnea to hemodynamic collapse and sudden death. This variability complicates early recognition and appropriate triage [3,4,5]. Risk stratification tools, such as the Pulmonary Embolism Severity Index (PESI) and simplified PESI (sPESI), have been developed to predict short-term mortality and guide management strategies. However, there is ongoing debate about additional clinical predictors that could improve the accuracy of these models, especially in identifying patients at high risk of early deterioration [6,7,8].

Syncope, defined as a transient loss of consciousness due to global cerebral hypoperfusion, could occur in patients with acute PE, though the reported prevalence varies across risk categories [5,9]. In patients with high-risk PE, syncope has been reported in 30–40% of cases, due to abrupt right ventricular failure and circulatory collapse [10]. Among patients with intermediate-to-high-risk PE, where right ventricular dysfunction exists without overt hypertension, syncope is less frequent: 10–25% of patients [6]. It is uncommon in intermediate-to-low-risk and low-risk PE [11].

The pathophysiology of syncope in PE is multifactorial: large embolic obstruction leads to acute right ventricular (RV) failure, impaired left ventricular filling, reduced cardiac output, and consequently cerebral hypoperfusion [12]. Right ventricular dysfunction in the setting of PE refers to the impairment of right ventricular performance due to increased pulmonary vascular resistance from thromboembolic obstruction. This event manifests as the inability of the RV to maintain adequate cardiac output and results in clinical signs such as hypotension, systemic hypoperfusion, and elevated jugular venous pressure [13].

Other mechanisms, such as arrhythmias triggered by RV strain, reflex-mediated vasodilation, or concomitant hypoxemia, may also contribute to this. Nonetheless, prodromal symptoms play an important role in differentiating the underlying causes of syncope. In patients with PE, syncope is preceded by acute dyspnea, palpitations, pleuritic chest pain and lightheadedness. Syncope in PE may arise without any identifiable trigger or may be provoked by postural change, exertion, couching, and other circumstances that increase right ventricular afterload or reduce venous return [14,15].

Importantly, syncope in the setting of PE often indicates a more severe hemodynamic compromise and has been proposed as a clinical marker of high-risk disease [16].

Several observational studies have investigated the prognostic significance of syncope in acute PE, with mixed results. Some have demonstrated a strong association between syncope and short-term mortality [11,17], while others suggested that the prognostic impact is attenuated once adjusted for hemodynamic instability and RV dysfunction [18,19]. The current European Society of Cardiology (ESC) guidelines recognize syncope as a possible indicator of severe PE but do not incorporate it as an independent variable in risk stratification scores [2]. Therefore, further investigation is warranted to clarify whether syncope can serve as an independent predictor of adverse outcomes in patients with PE.

The present study addresses this knowledge gap by retrospectively analyzing a cohort of 300 patients with confirmed PE. The primary objective was to determine whether syncope at presentation is an independent predictor of in-hospital mortality. Secondary objectives included assessing the prevalence of syncope, comparing clinical characteristics between patients with and without syncope, and identifying additional predictors of poor in-hospital outcomes.

## 2. Methods

### 2.1. Study Design and Population

We conducted a retrospective, single-center cohort study, including adult patients (≥18 years) admitted to the Clinical County Emergency Hospital Bihor, Department of Cardiology, with a confirmed diagnosis of acute pulmonary embolism (PE) between January 2022 and July 2025. The study was approved by the local institutional ethics committee. The study was conducted in accordance with institutional guidelines, and the requirement for individual informed consent was waived given the retrospective nature of the analysis.

### 2.2. Inclusion and Exclusion Criteria

Patients were eligible for the study if they had radiologically confirmed PE by computed tomography pulmonary angiography (CTPA) and complete medical records, including documentation of presenting symptoms, clinical findings, laboratory results, and outcomes.

Exclusion criteria were patients with syncope attributable to alternative causes unrelated to PE like seizures, hypoglycemia, or vasovagal events with no radiological evidence of PE, patients with incomplete clinical data preventing adequate risk stratification, and those with chronic thromboembolic pulmonary hypertension, in the last case to avoid confounding with acute PE presentations. Patients with a suspicion of PE and chronic or acute kidney disease were excluded too. They did not undergo CTPA because of the risk of worsening kidney failure.

### 2.3. Data Collection

Clinical and demographic variables were extracted from hospital records. The following information was collected: demographics, including age and sex; medical history, including cardiovascular risk factors, history of venous thromboembolism, malignancies, recent surgery, or immobilization; clinical presentation, including the presence of syncope at admission (defined as transient loss of consciousness with spontaneous recovery), dyspnea, chest pain, hemoptysis, and hemodynamic status; vital signs and laboratory data, including blood pressure, heart rate, oxygen saturation, troponin, and D-dimer; imaging findings, including right ventricular (RV) dysfunction on echocardiography (defined as RV/LV ratio >1.0, hypokinesis, or McConnell’s sign); treatment received, including anticoagulation regimen and systemic thrombolysis; and outcomes, including admission to intensive care unit (ICU), length of hospital stay, and in-hospital mortality.

### 2.4. Definition of Syncope

Syncope was defined as a sudden, transient loss of consciousness and postural tone, with spontaneous recovery, presumed to be due to global cerebral hypoperfusion. The diagnosis was based on documentation in medical records at admission and was confirmed by the attending physician after excluding seizures or metabolic causes [20].

### 2.5. Statistical Analysis

Data were analyzed using IBM SPSS Statistics version 26.0 (IBM Corp., Armonk, NY, USA).

Descriptive statistics were used. Continuous variables were tested for normality using the Shapiro–Wilk test. Normally distributed variables were expressed as mean ± standard deviation (SD), and non-normally distributed variables were expressed as median (interquartile range, IQR). Categorical variables were presented as frequencies and percentages.

Comparisons between groups were performed. Patients were divided into two groups (with syncope vs. without syncope). Continuous variables were compared using the independent *t*-test or Mann–Whitney U test, as appropriate. Categorical variables were compared using Chi-square or Fisher’s exact test.

Regression analysis was used. Univariate logistic regression was performed to assess the association between syncope and in-hospital mortality. Variables with *p* < 0.10 in univariate analysis were entered into a multivariate logistic regression model to identify independent predictors. Size effects were also determined. Odds ratios (OR) with 95% confidence intervals (CIs) were reported.

Regarding the significance threshold, a two-tailed *p*-value < 0.05 was considered statistically significant.

Cases with incomplete critical variables were excluded (listwise deletion).

## 3. Results

### 3.1. Study Population

A total of 300 patients with confirmed acute pulmonary embolism (PE) were included in the final analysis. The mean age of the study cohort was 64 ± 14 years, and 52% were female. The most common comorbidities were arterial hypertension (45%), diabetes mellitus (21%), active malignancy (15%), and previous venous thromboembolism (13%).

### 3.2. Prevalence of Syncope

Syncope at presentation was documented in 48 patients (16%). Compared to those without syncope, these patients were slightly older (67 ± 13 vs. 63 ± 14 years, *p* = 0.06) and more frequently had a history of cardiovascular disease (33% vs. 21%, *p* = 0.04).

The baseline characteristics of patients enrolled in the study are shown in Table 1.

### 3.3. Clinical Characteristics

Patients presenting with syncope had significantly higher rates of right ventricular (RV) dysfunction on echocardiography (33 patients with syncope and 82 patients without, 68.7% vs. 32.5%, *p* = 0.002), elevated troponin levels (36 patients with syncope and 111 without, 75% vs. 44%, *p* = 0.01), hypotension at admission (Systolic Blood Pressure (SBP) < 90 mmHg) (14 patients with syncope and 20 without, 29% vs. 8%, *p* < 0.001), and admission to an intensive care unit (ICU) (27 patients with syncope and 55 without, 56% vs. 22%, *p* < 0.001). Thrombolysis was performed in 9 patients with syncope and in 21 patients without syncope (19% vs. 8%, *p* = 0.03). These data are shown in Table 2, together with therapeutic options regarding thrombolysis.

All patients received anticoagulant therapy either with dose-adjusted enoxaparin or unfractionated heparin. Risk stratification using the PESI revealed that patients with syncope had higher risk classes compared to those without syncope. Patients with syncope mostly fell into Classes IV–V (high-to-very high risk), while those without syncope were in Classes III–IV (intermediate-to-high risk). These results align with the observed higher rates of ventricular dysfunction, elevated troponin levels, hypotension, ICU admissions, and thrombolysis, highlighting syncope as a marker of more severe PE. Patients with high-risk PE, according to PESI score (Pulmonary Embolism Severity Index), received thrombolytic therapy. Thrombolysis was performed in those with hemodynamic instability, according to guidelines and the institutional treatment protocol; there were no bleeding complications in our cohort.

**Figure 1:**
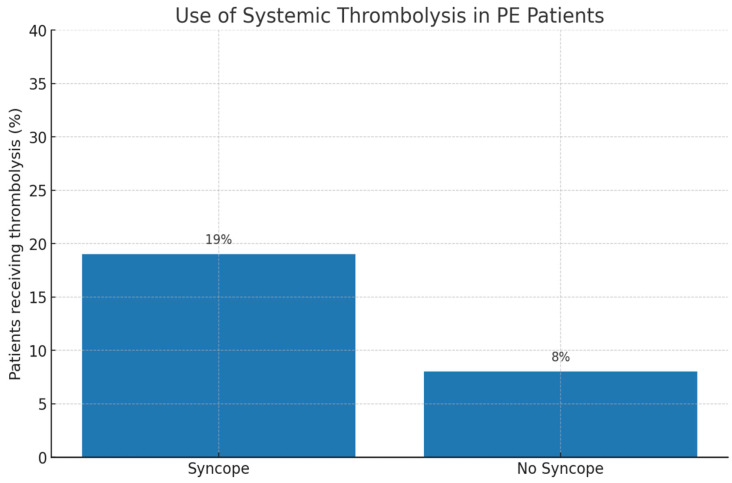
Use of systemic thrombolysis in PE patients—showing a higher rate in those with syncope (19% vs. 8%).

The use of systemic thrombolysis in our study is depicted in Figure 1.

### 3.4. In-Hospital Mortality

The overall in-hospital mortality rate was 12% (36 patients).

**Figure 2:**
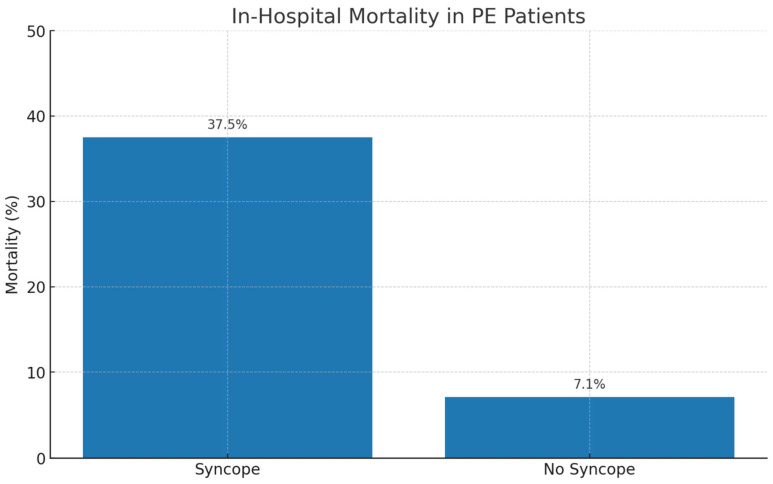
In-hospital mortality in patients with and without syncope.

Out of 48 patients with syncope, 18 (37.5%) died. From the group of 252 patients without syncope, 18 (7.1%) died. This difference was highly significant (*p* < 0.001), as shown in Figure 2.

**Figure 3:**
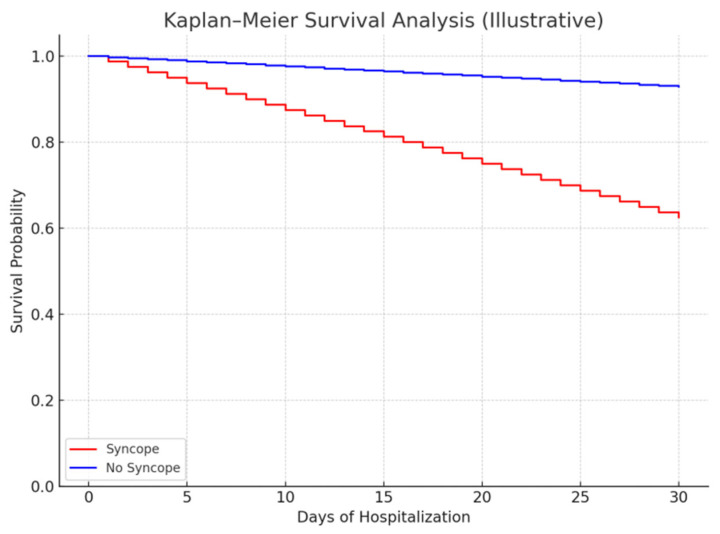
Kaplan–Meier survival curve shows a lower survival probability for patients with syncope compared to those without.

Additionally, Kaplan–Meier survival analysis demonstrated significantly lower survival probability in patients with syncope (log-rank *p* < 0.001), as shown in Figure 3.

### 3.5. Multivariate Analysis

In univariate logistic regression, factors associated with in-hospital mortality included syncope at presentation (OR 7.2, 95% CI 3.3–14.8, *p* < 0.001), right ventricular dysfunction (OR 3.9, 95% CI 2.0–7.5, *p* < 0.001), elevated troponin (OR 2.8, 95% CI 1.4–5.6, *p* = 0.003), and hypotension (OR 5.1, 95% CI 2.2–11.9, *p* < 0.001).

**Figure 4:**
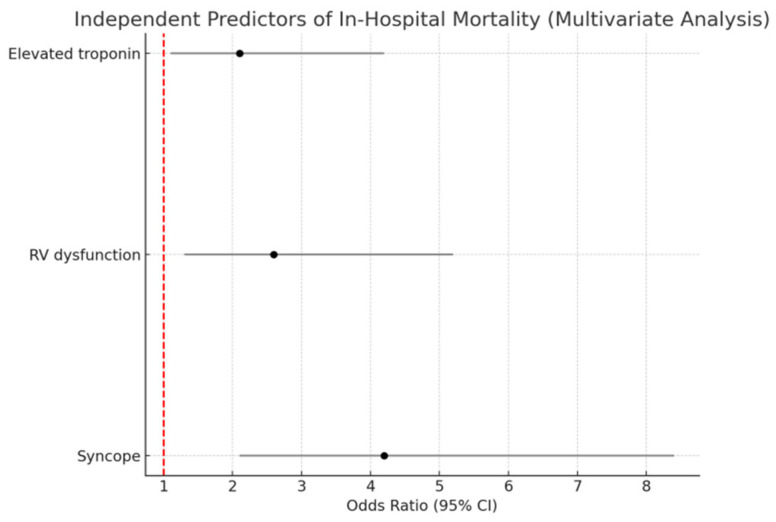
Forest plot visualizes the independent predictors of in-hospital mortality.

After multivariate adjustment, the following remained independent predictors of in-hospital mortality: syncope at presentation (OR 4.2, 95% CI 2.1–8.4, *p* < 0.001), right ventricular dysfunction (OR 2.6, 95% CI 1.3–5.2, *p* = 0.008), and elevated troponin (OR 2.1, 95% CI 1.1–4.2, *p* = 0.03). These data are depicted in Figure 4.

Multivariate analysis is shown in Table 3.

For patients with syncope versus patients without syncope, the effect sizes are given below. Among patients with syncope, 18 out of 48 (37.5%) died during hospitalization, compared to 18 out of 252 (7.1%) patients without syncope. This corresponds to a risk ratio of 5.25 (95% CI, 2.95–9.34), indicating that patients presenting with syncope had more than a fivefold higher risk of in-hospital mortality. The unadjusted odds ratio was 7.80 (95% CI, 3.66–16.61), while the multivariable model yielded an adjusted odds ratio of 4.2 (95% CI, 2.1–8.4). The absolute risk difference was +30.4% (95% CI +16.3% to +44.4%), corresponding to a number needed to harm (NNH) of 3.3. Standardized effect size measures supported these findings with Cohen’s h of 0.78 (large effect) and a Phi coefficient of 0.34 (moderate association). These results are illustrated in Table 4.

Summary of the key findings of our study:

- Syncope occurred in 16% of patients with acute PE.
- Patients with syncope had more severe clinical and echocardiographic features.
- Inhospital mortality was significantly higher in patients with syncope (37.5% vs. 7.1%).
- Syncope remained an independent predictor of mortality after adjustment for RV dysfunction and troponin levels.

## 4. Discussion

The present retrospective study of 300 patients with confirmed acute pulmonary embolism (PE) demonstrates that syncope at presentation is a strong, independent predictor of in-hospital mortality. Patients with syncope were more likely to present with right ventricular (RV) dysfunction, elevated troponin, hypotension, and ICU admission, reflecting a more severe hemodynamic profile. Importantly, the association between syncope and mortality remained significant even after adjustment for established prognostic factors, suggesting that syncope itself carries distinct prognostic value in acute PE.

### 4.1. Syncope as a Marker of Hemodynamic Instability

Syncope occurs in approximately 10–20% of patients with acute PE, consistent with our observed prevalence of 16% [1,2]. The pathophysiological mechanisms are multifactorial but primarily involve acute RV failure secondary to large embolic burden, leading to reduced left ventricular preload and global cerebral hypoperfusion. Reflex-mediated vasodilation and arrhythmias may also contribute [6]. Our findings that syncope was associated with RV dysfunction, elevated troponin, and hypotension further support this mechanism, underscoring syncope as a clinical marker of hemodynamic compromise.

### 4.2. Treatment Considerations in Acute Pulmonary Embolism with Syncope

The cornerstone of therapy in acute pulmonary embolism (PE) is prompt initiation of anticoagulation, which remains the standard of care across all risk categories. Low-molecular-weight heparin (LMWH), fondaparinux, and direct oral anticoagulants (DOACs) are increasingly preferred due to predictable pharmacokinetics and ease of administration [1]. Unfractionated heparin (UFH) is reserved for patients in whom rapid reversal may be necessary, particularly when systemic thrombolysis or invasive interventions are considered. Nevertheless, patients with pulmonary embolism that present with syncope on admission should be monitored closely as it may necessitate escalation of therapy [1,11,17].

In our cohort, anticoagulation was universally initiated, consistent with guideline-directed management. However, patients presenting with syncope more frequently required escalation of therapy. Specifically, systemic thrombolysis was administered in 19% of patients with syncope compared to 8% without syncope (*p* = 0.03). This observation reflects the fact that syncope at presentation was strongly associated with markers of hemodynamic compromise, including hypotension, right ventricular dysfunction, and myocardial injury.

Systemic thrombolysis, typically with intravenous alteplase (100 mg over 2 h), is the recommended treatment for patients with high-risk (massive) PE, defined by sustained hypotension or shock. In intermediate-to-high-risk PE, defined by right ventricular dysfunction and elevated troponin, thrombolysis may be considered in cases of clinical deterioration despite anticoagulation. Notably, reduced-dose regimens (e.g., alteplase 50 mg) have been explored to mitigate bleeding risk, particularly in elderly patients or those with comorbidities. For patients with contraindication to thrombolysis, catheter-directed therapy or surgical embolectomy could be an option [1,21,22,23].

The higher thrombolysis rate in patients with syncope in our study likely reflects the perception of syncope as a clinical surrogate of hemodynamic instability, prompting more aggressive treatment strategies. This is supported by the markedly higher in-hospital mortality observed in this group (37.5% vs. 7.1%). Our findings underscore the need to carefully evaluate patients with syncope for early markers of severity and to individualize treatment strategies, balancing the benefits of reperfusion therapy against the risks of bleeding.

### 4.3. Comparison with Previous Studies

Our results align with prior studies that reported higher short-term mortality in patients with PE presenting with syncope. Iqbar et al. [16] remarked that hypotension at admission was more frequent in patients with syncope compared to those without syncope, as was increased mortality. In contrast, some investigators suggested that the prognostic significance of syncope is attenuated after adjusting for RV dysfunction or shock [18]. The persistence of syncope as an independent predictor in our multivariate model suggests that it may provide incremental prognostic information beyond traditional markers.

Roncon et al. [24] noticed that in 26.6% of patients syncope was the initial manifestation of the disease, and mortality was markedly higher in these patients compared to patients without syncope at admission (42.5% to 6.2%).

Additionally, Barco et al. [17] showed that patients with syncope at admission had a significantly higher prevalence of hemodynamic instability and right ventricle dysfunction (RVD). In addition, higher early death prevalence was observed (73% higher odds of dying within 30 days compared to those without syncope).

Keller et al. [11] found syncope to be associated with increased mortality, particularly in hemodynamically unstable patients. Furthermore, mortality was lowered by systemic thrombolysis. The same conclusion was reported by De Winter et al. [25] in a meta-analysis.

Zhang et al. [15] showed that syncope was associated with symptoms like chest pain, palpitations, elevated troponin, and RVD. Hemodynamic instability was associated with increased in-hospital death.

Seghda et al. [18] reported that thrombolysis was significantly more often performed in patients with syncope. Furthermore, dilatation of the right ventricle chamber was noted in patients presenting with syncope at admission.

Overall, the results of our study corroborate previous studies, emphasizing that syncope in patients with pulmonary embolism can be associated with hemodynamic compromise, right ventricular dysfunction, and increased short-term mortality.

### 4.4. Clinical Implications

The recognition of syncope as a “red flag” symptom has important clinical implications. One of them is risk stratification, because syncope may be considered for integration into existing prognostic tools such as PESI or sPESI, potentially improving their ability to identify high-risk patients [17,26,27]. The presence of syncope is important in triage decisions too. Patients with PE and syncope should be promptly evaluated for RV dysfunction and hemodynamic instability and monitored in higher-acuity settings, such as intensive care units [6,11].

Some particular therapeutic considerations should be made for patients with PE and syncope. The presence of syncope may support early consideration of aggressive therapies, including systemic thrombolysis or catheter-directed interventions, especially when accompanied by RV dysfunction [1,24].

Syncope is important in the evaluation of patients because of its diagnostic implications: it is often attributed to benign causes such as vasovagal events, especially in elderly patients. Awareness that syncope may signal severe PE could prevent delays in diagnosis and treatment [6,28,29].

## 5. Strengths and Limitations

The strengths of our study include the relatively large cohort size and the detailed comparison of clinical, laboratory, and echocardiographic parameters between patients with and without syncope. The use of multivariate regression allowed us to adjust for potential confounders and confirm the independent association of syncope with mortality.

However, several limitations must be acknowledged. First, the retrospective design is inherently prone to selection and information bias. Syncope may have been underreported or misclassified, as not all episodes were observed by healthcare personnel. Second, our study was conducted in a single center, which may limit generalizability. Third, we did not assess long-term outcomes beyond hospital discharge, so the prognostic impact of syncope on medium- or long-term survival remains unknown. Finally, while we adjusted for major prognostic markers, residual confounding cannot be entirely excluded.

## 6. Conclusions

This retrospective analysis of 300 patients with confirmed acute pulmonary embolism (PE) demonstrates that syncope at presentation is a strong and independent predictor of in-hospital mortality. Patients who experienced syncope had markedly higher mortality compared with those without and were more likely to present with right ventricular dysfunction, elevated troponin, and hypotension—all markers of hemodynamic compromise. Importantly, syncope retained its prognostic significance even after multivariate adjustment, underscoring its value as a simple but powerful clinical indicator.

Our findings reinforce the concept that syncope in the context of PE is not a benign manifestation but rather a “red flag” symptom that should immediately prompt clinicians to consider high-risk disease. Recognition of syncope as a prognostic marker may aid in early risk stratification, complementing established tools such as PESI and sPESI; triage decisions, supporting ICU admission or closer monitoring; and therapeutic planning, where syncope may tip the balance toward aggressive reperfusion therapies in selected patients.

However, the retrospective, single-center design and limited sample size restrict the generalizability of our results, and residual confounding cannot be excluded. Larger, prospective, multicenter studies are warranted to validate these observations, assess long-term outcomes and clarify whether incorporating syncope into risk stratification or treatment algorithms can improve patient care.

In conclusion, syncope should be recognized as a critical warning sign in patients with acute PE. Incorporating syncope into clinical decision-making algorithms may enhance early recognition of high-risk patients and facilitate timely, life-saving interventions.

## 7. Future Directions

Future prospective, multicenter studies are warranted to validate our findings and clarify the incremental value of syncope in risk stratification models. Incorporating syncope into decision algorithms may help refine the identification of patients who require intensive monitoring and advanced therapies. Additionally, further research should explore whether syncope-guided therapeutic strategies improve outcomes, for example, by prompting earlier use of reperfusion therapies in patients with otherwise borderline indications.

In summary, our study highlights syncope as an important clinical sign in the context of acute PE, independently associated with higher in-hospital mortality. Syncope should be recognized not merely as a presenting symptom but as a warning signal of severe disease, warranting aggressive diagnostic and therapeutic measures.

## Figures and Tables

**Table 1:** Baseline characteristics of patients with and without syncope.

Characteristic	Syncope (n = 48)	No Syncope (n = 252)	*p*-Value
Age, years	67 ± 13	63 ± 14	0.06
Female, n (%)	24 (50%)	132 (52%)	0.78
Arterial hypertension, n (%)	22 (46%)	112 (44%)	0.78
Diabetes mellitus, n (%)	11 (23%)	52 (21%)	0.72
Active malignancy, n (%)	8 (17%)	37 (15%)	0.68
Previous Venous Thromboembolism (VTE), n (%)	6 (13%)	33 (13%)	0.95
Cardiovascular disease, n (%)	16 (33%)	53 (21%)	0.04

**Table 2:** Clinical, laboratory, and therapeutic findings in patients with and without syncope.

Parameter	Syncope (n = 48)	No Syncope (n = 252)	*p*-Value
SBP < 90 mmHg, n (%)	14 (29%)	20 (8%)	<0.001
Heart rate, bpm	104 ± 22	96 ± 18	0.02
RV dysfunction on echocardiography, n (%)	33 (68.7%)	82 (32.5%)	0.002
Elevated troponin, n (%)	36 (75%)	111 (44%)	0.01
D-dimer, ng/mL	2450 ± 1100	2200 ± 1050	0.18
ICU admission, n (%)	27 (56%)	55 (22%)	<0.001
Thrombolysis, n (%)	9 (19%)	21 (8%)	0.03

**Table 3:** Logistic regression analysis for predictors of in-hospital mortality.

Variable	Univariate OR (95% CI)	*p*-Value	Multivariate OR (95% CI)	*p*-Value
Syncope at presentation	7.2 (3.3–14.8)	<0.001	4.2 (2.1–8.4)	<0.001
RV dysfunction	3.9 (2.0–7.5)	<0.001	2.6 (1.3–5.2)	0.008
Elevated troponin	2.8 (1.4–5.6)	0.003	2.1 (1.1–4.2)	0.03
Hypotension (SBP < 90 mmHg)	5.1 (2.2–11.9)	<0.001	-	-

**Table 4:** Outcomes according to syncope presentation with effect sizes.

Outcome	Risk (Syncope)	Risk (No Syncope)	Risk Ratio	Odds Ratio	Risk Difference	Cohen’s H	Phi (φ)
In-hospital mortality	37.5%	7.1%	5.25	7.8	30.4%	0.78	0.34
ICU admission	25.0%	8.3%	3	3.67	16.7%	0.46	0.2
Thrombolysis	35.4%	10.7%	3.31	4.57	24.7%	0.61	0.26
Hypotension	47.9%	20.6%	2.32	3.54	27.3%	0.59	0.23
RV dysfunction	83.3%	34.5%	2.41	9.48	48.8%	1.04	0.36

## Data Availability

The raw data supporting the conclusions of this article will be made available by the authors on request.

## Abbreviations

Abbreviation	Full Form
PE	Pulmonary Embolism
RV	Right Ventricle
RVD	Right Ventricular Dysfunction
ICU	Intensive Care Unit
SBP	Systolic Blood Pressure
VTE	Venous Thromboembolism
LMWH	Low-Molecular-Weight Heparin
UFH	Unfractionated Heparin
DOACs	Direct Oral Anticoagulants
CTPA	Computed Tomography Pulmonary Angiography
PESI	Pulmonary Embolism Severity Index
sPESI	Simplified Pulmonary Embolism Severity Index
OR	Odds Ratio
CI	Confidence Interval
